# Supplementary material for: A comparison of educational events for physicians and nurses in Australia sponsored by opioid manufacturers
Source: PLoS One. 2021 Mar 18;16(3):e0248238. doi: 10.1371/journal.pone.0248238 (PMC7971255; doi:10.1371/journal.pone.0248238)
Supplement: S1 Table — (DOCX) [file pone.0248238.s001.docx]

**S1 Table. Variables of interest and keyword search strategy for coding**

| **Variable** | **Column** | **Keywords** | **Y/N** | **Coding** |
| --- | --- | --- | --- | --- |
| Total_cost | Total cost of function | N/A |  | Continuous; AUD |
| Total_cost_food | Total cost of hospitality | N/A |  | Continuous; AUD |
| Pain_related | Description of function | Pain; opioid; opiate; narcotic; analges*; Morphine; fentanyl; hydromorphone; buprenorphine; naloxone; oxycodone; codeine; tramadol; Tapentadol; Tramal; Jurnista; Durogesic; Norspan; Sevredol; MS Mono; Dilaudid; Targin; OxyNorm; OxyContin; Endone; Palexia; Contin; Narcan; Prodeine; Panadeine; Suboxone | Y | 1=Y  0=N |
| Pain_related (secondary terms) | Description of function | Neuropathic; nerve; palliati*; addict*; overdose; harm reduction | Y, if unambiguous | 1=Y  0=N |
| Pain_related  (Mundipharma) | Description of function | All Mundipharma events are coded as pain-related unless specifically stating otherwise (see exclusion keyword searches below) | Y | 1=Y  0=N |
| Pain_related  (coding for NOT opioid-related pain) | Description of function | Chest; neuropathy; neuropathies | N | [blank]=N  0=Y |
| Pain_related  (coding for NOT pain-related based on company product listings for specific conditions) | Description of function  Professional status of attendees | For Mundipharma:  Respiratory; asthma; diabetes; pancrea*; ophthalmol*; menopause; osteoporosis; gastro*; endoscop*; bowel; prostate cancer; hemaetol*; lymphoma; chemotherapy; nausea; radiology; radiation  For Janssen: ankylosing spondylitis | N | [blank]=N  0=Y |
| PROFESSION | Coded profession variables |  |  | 1=Y prescriber_only  2= Y any_nurse  3= Y only_nurse  4= Y other health care professionals (no nurse, no physician)  5= Y prescriber and other HCP (no nurse) |
| Prescriber_present | Professional status of attendees | GP; general practitioner; family medicine; Registrar; resident; intern; RMO; resident medical officer; JHO; SHO; senior house officer; PHO; principal house officer; fellow; Specialist; consultant; senior medical officer; SMO; visiting medical officer; VMO; general medicine; general physician; *ology physician; *ology doctor;  allergist; allergy anesthesiologist; anesthetist; anaesthetist; anaesthesiologist; andrologist; cardiologist; dermatologist, diabetologist; emergency physician; emergency medicine physician; endocrinologist; epileptologist; gastroenterologist; geriatrician; getriatric physician; gynaecologist; obstetrician; OB/GYN; haematologist; hematologist; hepatologist; immunologist; infectious disease physician; infectious disease doctor; internal medicine physician; microbiologist; neonatologist; neurologist; nuclear medicine physician; nephrologist; renal physician; renal doctor; urologist; oncologist; ophthalmologist; pharmacologist; psychogeriatrician; rheumatologist; radiologist; pulmonologist; respiratory physician; respiratory medicine physician; respiratory medicine doctor; palliative care physician; pathologist; sexual health physician; sexual health doctor; psychiatrist; psychiatry doctor; paediatrician; surgeon; surgery doctor; intensive care doctor; intensivist; intensive care physician; cardiothoracic; advanced trainee; nurse practitioner; NP; nurse prescriber | Y | 1=Y  0=N |
| Only_prescriber  [Only prescribers in attendance] | Professional status of attendees Description of function | See above keywords; no other professions in attendance | Y | 1=Y  0=N |
| Any_nurse  [At least one nurse in attendance] | Professional status of attendees Description of function | Nurse; nurses; midwife; midwives; nurse anaesthetist; clinical nurse specialist | Y | 1=Y  0=N |
| Only_nurses  [Only nurses in attendance] | Professional status of attendees Description of function | Nurse; nurses; midwife; midwives; nurse anaesthetist; clinical nurse specialist | Y | 1=Y  0=N |
| Clinical_setting | Venue | Health; hospital; clinic; practice; medicare local; health centre; health district; surgery; medical centre; medical; health care centre; healthcare centre; specialist centre; cancer centre; cancer care centre; heart centre; medical and dental centre; endocrine centre; radiotherapy centre; radiation centre; optical centre; eye centre; renal unit; ward; department; dept; community health; family planning; education centre; general practice | Y | 1=Y  0=N |
| TYPE | Description of function |  | |  |
| Type_journalclub |  | Journal club; journalclub | Y | 1=Y  0=N |
| Type_training |  | Workshop; Inservice; in-service; in?service^¶^ | Y | 1=Y  0=N |
| Type_grandrounds |  | Grand round; grandround | Y | 1=Y  0=N |
| Type_conference |  | scientific meeting; scientific weekend; annual meeting; congress; conference; symposium | Y | 1=Y  0=N |
| Type_meeting^^ |  | departmental meeting; case review; case conference; case study meeting; case study conference; multidisciplinary meeting; educational meeting; dinner meeting; lunch meeting | Y | 1=Y  0=N |
| Type_Product_Launch |  | Product launch; launch; OR name of branded or generic drug | Y | 1=Y  0=N |

*Truncated term

^†^ All Mundipharma-related events were then coded as “pain-related” unless the event explicitly stated otherwise.

^^Excludes “meeting” types listed under type_conference
