# Supplementary material for: A comparison of educational events for physicians and nurses in Australia sponsored by opioid manufacturers
Source: PLoS One. 2021 Mar 18;16(3):e0248238. doi: 10.1371/journal.pone.0248238 (PMC7971255; doi:10.1371/journal.pone.0248238)
Supplement: S2 Table — (DOCX) [file pone.0248238.s002.docx]

**S2 Table. Coding manual for content analysis**

| **Variable** | **Column searched** | **Notes** | **Coding** |
| --- | --- | --- | --- |
| No_information | Description of function  Professional status of attendees  Venue | Does not mention any content beyond event format (e.g. journal club, grand rounds) and takes place at a full service hospital, clinic or non-clinical setting | 1=Y  0=N |
| Pain_generic | Description of function  Professional status of attendees  Venue | States only keywords for “pain” (e.g. “pain,” “analgesia”); multidisciplinary meetings (multiple specialties present); no specific pain type indicated OR names a specific general practice | 1=Y  0=N |
| Pain_acute | Description of function  Venue | Mentions acute and or surgical/surgery, injury, fracture, post-operative, perioperative; sports injury; sport (s) medicine; trauma; emergency; emergency department; emergency medicine | 1=Y  0=N |
| Pain_chronic | Description of function  Venue | Mentions chronic or ongoing chronic health condition; “persistent” pain; back pain/care; arthritis; osteoarthritis; OA; spinal care; rheumatol*; rehab*; musculoskeletal;  non-cancer; non cancer, non- cancer | 1=Y  0=N |
| Pain_cancer | Description of function  Venue | Mentions cancer; palliation; palliative; end of life care; advanced care; oncology; hospice; OR names a specific cancer hospital, palliative care or hospice facility | 1=Y  0=N |
| Pain_geriatric | Description of function  Venue | Mentions geriatrics; gerontology; aged care; age care; long term care; dementia; ageing; elderly; older adults; OR names a specific aged care facility | 1=Y  0=N |
| Pain_nerve | Description of function  Venue | Mentions nerve pain; neuropathic pain | 1=Y  0=N |
| Pain_addiction | Description of function  Venue | Mentions addiction; harm reduction; dependence or dependent; tolerance or tolerant; “opioid users”; “drug problems” OR names a specific addiction medicine clinic | 1=Y  0=N |
| Pain_adverse | Description of function | Mentions adverse effects (other than addiction, dependence, or tolerance) such as constipation; overdose; “safety”; side-effects; side effect; renal impairment; overprescribe*; risk management | 1=Y  0=N |
| Pain_nonpharm | Description of function  Venue | Mentions non-pharmacological modalities (acupuncture; psychosocial; mindfulness; spiritual) | 1=Y  0=N |
| Drug_named | Description of function | morphine; fentanyl; hydromorphone; buprenorphine; naloxone; oxycodone; codeine; tramadol; Tapentadol; Tramal; Jurnista; Durogesic; Norspan; Sevredol; MS Mono; Dilaudid; Targin; OxyNorm; OxyContin; Endone; Palexia; Contin; Narcan; Prodeine; Panadeine; Suboxone | 1=Y  0=N |
| Branded_drug | Description of function | Tapentadol; Tramal; Jurnista; Durogesic; Norspan; Sevredol; MS Mono; Dilaudid; Targin; OxyNorm; OxyContin; Endone; Palexia; Contin; Narcan; Prodeine; Panadeine; Suboxone | 1=Y  0=N |
