# Supplementary material for: A comparison of educational events for physicians and nurses in Australia sponsored by opioid manufacturers
Source: PLoS One. 2021 Mar 18;16(3):e0248238. doi: 10.1371/journal.pone.0248238 (PMC7971255; doi:10.1371/journal.pone.0248238)
Supplement: Attachment — Submitted filename: Response to reviewers PLOS One 26.01.21.docx [file pone.0248238.s003.docx]

Quinn Grundy, PhD RN

Assistant Professor

Faculty of Nursing

University of Toronto, ON, Canada

E-mail: [quinn.grundy@utoronto.ca](mailto:quinn.grundy@utoronto.ca)

Tim Mathes

Academic Editor

*PLOS One*

January 25, 2021

Re: Submission of revised manuscript (PONE-D-20-20905) entitled, “A comparison of educational events for physicians and nurses in Australia sponsored by opioid manufacturers”

Dear Dr. Mathes,

Thank you so much for these constructive reviews and the opportunity to revise the above named manuscript. In our response, we have provided, point by point, our replies to the comments made by the reviewers, explaining how we have dealt with them in the manuscript.

We have updated the conflict of interest statement per the journal’s guidelines. It now reads,

“The authors have no conflicts of interest. Quinn Grundy currently serves as Academic Editor for PLOS ONE. This does not alter our adherence to  PLOS ONE policies on sharing data and materials.”

Thank you for your time in consideration of this revision.

Sincerely,

Quinn Grundy

on behalf of

Sasha Mazzarello

Sarah Brennunstuhl

Emily Karanges

| **Editors’ comments** | **Response** | **Relevant changes** |
| --- | --- | --- |
| A premise for acceptance is adequate consideration of the issues raised regarding data coding/analysis and characteristics of events. | Thank you for the opportunity to revise this manuscript. We have responded to each of the comments and made corresponding changes to the manuscript, which are noted below. | See below |
| **Reviewers’ comments** | **Response** | **Relevant changes** |
| **Reviewer 1** | | |
| This study addressed an important issue of industry promotional activity in the medical field; clarifying the current situation among opioid manufacturer-sponsored educational events in Australia. The study also focused on the differences between pain-related educational events for physician and those for nurses. This is a cross sectional study with numerous data, the manuscript is well structured and the methodology and analysis seems to be appropriate. I have added some following comments. | Thank you for your encouraging and constructive comments. We respond to each of your comments below. | N/A |
| The authors describe in the "Coding" section, "We coded all Mundipharma events as "pain-related" unless the event explicitly stated otherwise, given the company's limited product profile."  I understand that Mundipharma's main products are pain-related drugs, but the profile may vary from country to country. Do you have the data to support this? | On Mundipharma’s Australian website, Mundipharma lists 8 product categories available on the Australian market: First aid, Drug free pain relief, Medication lubricant, Overdose reversal, Ophthalmology, Oncology, Respiratory, and Pain.  However, the only products that could be associated with educational events sponsored in the data set during the study period were those with the Pain, Oncology, and Respiratory product categories. Of Mundipharma’s listing, the only products on the market prior to September 2018 were:  palonosetron (Aloxi injection)  pralatrexate (Folotyn injection)  netupitant and palonosetron (AKYNZEO®)  fluticasone propionate / eformoterol (Flutiform)  All products in the First aid, Drug free pain relief, Medication lubricant product categories are available over-the-counter and thus, are not covered by the transparency reporting requirements and would not be associated with any events reported in this dataset.  In the Overdose reversal category, Mundipharma markets Nyxoid (naloxone), however, this intranasal spray formulation was not available on the Australia market during the study period (the Therapeutic Goods Administration entered Nyxoid into the register after September 2018). It is also available as a pharmacist-only medication (Schedule 4) and does not require a prescription, thus, would not be in the transparency dataset.  In contrast, Mundipharma’s portfolio during the study period included 6 different opioids that were under patent protection and approved for the Australia market and for government subsidy during the study period (see Table 1 in the manuscript). Given that Mundipharma is 100% owned by the Sackler Family, which also owns Purdue Pharma, and the global promotion of opioids undertaken by these companies is well-documented, we believed it reasonable to assume that events were pain-related in the absence of other information.  To validate this, we explicitly excluded all events that could be related to other product offerings, by coding the event description and professional status of attendees according to the following keywords related to the products and their indications (see Supplementary Table 1):  Respiratory; asthma; diabetes; pancrea*; ophthalmol*; menopause; osteoporosis; gastro*; endoscop*; bowel; prostate cancer; hemaetol*; lymphoma; chemotherapy; nausea; radiology; radiation  There were 1197 events sponsored by Mundipharma that did not provide any details about the content of the event (e.g. 1 hour Journal club, held at a restaurant, attended by Anaesthetists, Nurses, Registrars). We have coded these as pain-related based on the above assumptions about Mundipharma’s portfolio and marketing activities, however, this may mean that we have overestimated the number of pain-related educational events sponsored by Mundipharma. We have added some discussion of this to the limitations section.  We have clarified our coding methodology in the Methods section to incorporate some of this detail. | The revised text in the Methods under “Coding” reads,  “We coded all Mundipharma events as “pain-related” unless the event explicitly stated otherwise, given the company’s limited product profile and the dominance of opioid medications within this portfolio. We further assumed that events were “pain-related” in the absence of details about the event content (e.g. 1 hour Journal club) given that Mundipharma is a member of the global network of Mundipharma companies (which are 100% owned by the Sackler family, the owners of Purdue Pharma); Mundipharma’s aggressive promotion of opioid medications and particularly oxycodone internationally is well-documented ^1^. We conducted additional keyword searches to exclude events as “not pain-related” based on Mundipharma’s non-opioid product offerings, which at the time of the study, included respiratory products for asthma, diabetes products, and oncology products related to lymphoma and anti-nausea agents (see Supplementary Table 1).”  We have added text to the Limitations section, which reads,  “We may have overestimated the number of events that were “pain-related” and sponsored by Mundipharma in assuming that events were pain-related in the absence of details about the event content or attendee specialty. However, given that Mundipharma’s product portfolio is dominated by pain-related medications and the promotion of opioid medications by Mundipharma companies both overtly and covertly is well-documented, we believe this was a reasonable assumption ^1^.” |
| Was there any difference in the details of the events between the three companies? | The level of details of the events varied both between companies, but also within a single company’s reports. Given this variability, there were no appreciable differences in reporting that could be attributed to differences between companies.  In some cases, the description of the function contained the name of the drug, the topic of the seminar and its duration (e.g. Janssen Educational Event Series SR Hydromorphone. A once a day oral opioid for chronic pain management - 1 hr). On the other side of the spectrum, the description of the function was sparse and simply stated the event type and duration (e.g. Journal club – 1 hour duration). For all companies, however, the description of the function was accompanied by details on the venue (enabling determination of whether the event was held in a clinical setting or not) and the types and specialties of health professionals attending (which in some cases allowed us to infer the focus, e.g. drug and alcohol specialists, palliative care physicians, or aged care nurses).  We have added some details to the Results section to describe the variability in the reporting. We also note here that there was insufficient information to determine the focus of the event for 32% of the sample.  We note in the limitations section some of the challenges associated with this particular data set.  Because the number of pain-related events sponsored by each company varied so widely (15 for bioCSL to 3,260 for Mundipharma) and the sample was dominated by Mundipharma-sponsored events (96% of pain-related events) we could not determine any meaningful comparisons between the three companies. Particularly, the small sample of pain-related events sponsored by bioCSL (n=15) precluded any statistical comparisons. | See additional detail in the Results section in the paragraph first describing Table 2,  “For about a third of events (32%), manufacturers did not report sufficient information to determine the content focus, mentioning only the event type and duration of the event (e.g. “journal club. – 1 hour duration,” “departmental meeting – 2 hour duration”). For the remainder, the description of the event mentioned a topic (e.g. “Palliative care study day), provided the title of a presentation (e.g. “Reducing the burden of persistent pain”), mentioned a particular product (e.g. “Series SR Hydromorphone”), or the focus could be inferred by the venue (e.g. aged care facility) or specialty of professionals in attendance (e.g. orthopaedic surgeons).”  We have added a sentence to the limitations section,  “Further, there was a high degree of variability in terms of the specificity and comprehensiveness of reporting with insufficient detail to determine the content focus of about 1/3 of sampled events.” |
| While it is conceivable that pharmaceutical companies may do more promotional activities when there are competing drugs, opioids are promoted with a different aim to increase the target population, which has had the unfortunate consequence of inappropriate prescriptions. Most events in the present study were held by Mundipharma. Is the majority of pain-related prescriptions in Australia by Mundipharma? If so, do you see these efforts of Mundipharma as part of promotion to increase prescribing through education rather than competition with competing companies? | We appreciate the important point you are making here and believe this also justifies a focus on understanding promotion to nurses as a mechanism for expanding the settings and target populations for a particular drug. For example, nurses are a key audience to ensure increased assessment of pain, and increased uptake and compliance with pain medications as a means to increase the number of prescriptions written and filled. In the Introduction, we have revised the section on promotion to nurses to account for this important point.  Oxycodone (marketed by Mundipharma) accounted for 37% of all opioid prescriptions dispensed in 2016-2017 in Australia. We have added this information to the Introduction. This percentage likely increases when Mundipharma’s other opioid products are accounted for. | We have added a sentence to the introduction at the end of the 2nd paragraph,  “In Australia, in 2016-2017, oxycodone (marketed by Mundipharma Australia) accounted for 37% of the 15.4 million opioid prescriptions dispensed^2^.”  The section on promotion to nurses now reads,  “Through promotional campaigns, pharmaceutical companies seek not only to increase their market share, but also to expand their market through promoting awareness, screening, assessment of conditions for which the drug is indicated and expanding the indications for the drug, particularly to new settings and populations ^3^. Nurses play a key role in assessing pain, and administering opioid medications and particularly, medications prescribed ‘as needed,’ across multiple healthcare settings including acute care, primary care, and long term care. Nursing practice also attends to the multiple psychosocial factors that serve as determinants of pain and they play a large role in coordinating care, referrals, and the administration of alternative modalities for pain treatment ^4,5^. Thus, it is important to understand patterns in promotion targeted at nurses as well as prescribers, particularly as nurses’ relationships with industry have received less policy attention than physicians’.” |
| **Reviewer 2** | | |
| This article addresses a hugely important public health issue. It is well written and insightful. Thank you for the opportunity to review it. It does a great job of extending the field of research on industry payments beyond the US as well as beyond physicians. The findings and discussion are very clear. I only have a few questions regarding the introduction and methods. | Thank you for your encouraging comments – we have responded to each of your questions below. | N/A |
| It would be useful to see a bit more justification of why Australia is a key case to study the behaviour of opioid manufacturers. | We have significantly revised and restructured the Introduction to bring the focus on Australia up front. Primarily, we provide more evidence around the magnitude and severity of the public health harms associated with opioid use in Australia, which follows trends in the United States and Canada. Then, we describe in the introductory paragraphs the (globally) unique dataset available in Australia that allows for study of educational events sponsored by opioid manufacturers. We more clearly explain that this analysis is useful for understanding the roots of the Australian epidemic of opioid-related harms, but also for understanding sponsored education as a mechanism for public health harm more broadly. | The revised Introduction now reads,  “Opioid-related overdoses and deaths constitute a contemporary public health crisis in several countries including the United States, Canada and Australia. While the United States and Canada continue to lead in terms of prescribing and utilisation of opioid analgesics ^6^, and the burden of opioid-related mortality ^7^, Australia appears to be following a similar pattern ^8^. Between 1992 and 2012, the dispensing of opioid medications in Australia increased 15-fold, marked by an increase in the use of strong, long-acting and transdermal formulations ^9^; specifically, there was a major shift from the use of dextropropoxyphene and morphine in 1990, to oxycodone, tramadol and fentanyl in 2014 ^10^. During the same time period, opioid-related harms also increased including opioid-related hospitalizations and deaths due to accidental poisoning ^2,9^, which were attributed more often to prescription opioids than to heroin ^2^.  Through the 1990s and 2000s, opioid manufacturers aggressively promoted and heavily marketed opioid medications, particularly for chronic, non-cancer pain, despite a paucity of evidence on their safety and efficacy for long-term use ^11,12^. For example, Purdue Pharma introduced a sustained-release oxycodone preparation (OxyContin) in the United States in 1996, which quickly reached blockbuster status with combined sales of nearly $3 billion (over 14 million prescriptions) by 2002 ^11^. Compared with other available opioid preparations, Purdue Pharma’s formulation offered no clinical benefits; thus, the explosion in sales of oxycodone was attributed to the company’s aggressive promotional and marketing campaign ^11^. Since 2010, prescriptions for OxyContin began to fall in the United States; however, the owners of Purdue Pharma, the Sackler family, pursued similar marketing strategies internationally through a global network of companies, Mundipharma ^1^. In Australia, in 2016-2017, oxycodone (marketed by Mundipharma Australia) accounted for 37% of the 15.4 million opioid prescriptions dispensed ^2^.  However, there is relatively little research on the nature of opioid promotion outside of the United States. Journalists and researchers have primarily relied on industry documents made public through litigation (primarily in the United States) or databases of pharmaceutical industry payments to health professionals (such as the United States Open Payments database) to study the association between industry payments and opioid-related health outcomes ^13-15^.  In 2007, Australia was one of the first countries to move toward greater transparency of the relationships between health professionals and the pharmaceutical industry ^16^. From October 2011 to September 2015, Medicines Australia, the trade association for the prescription medicines industry in Australia, required member companies to report the details of sponsored events for all registered health professionals ^17^. This dataset, which is publicly available ([http://dx.doi.org/10.4227/11/592631edbd9d5](https://webmail.sydney.edu.au/owa/redir.aspx?C=Dblz1ojd2d3urn39BYSIsM0Xh6cQwxwhK9zEcaYRDcw4u57twrnUCA..&URL=http%3a%2f%2fdx.doi.org%2f10.4227%2f11%2f592631edbd9d5)), is unique globally in that it details sponsored educational events including all registered health professionals, whether or not they have prescribing authority, and, in addition to the dollar value of the sponsorship, contains a description of the event’s content, the hospitality provided, the venue, and the number and professional status of attendees. Thus, we aimed to analyse the nature of pain-related educational events sponsored by opioid manufacturers to understand patterns in relation to the content, setting, and to compare events targeting prescribers and non-prescribing nurses.” |
| In addition, it would be helpful to see more background on why nurses are key to target in opioid drug promotion in general and specifically in Australia. Is there any data on this? This would help international readers situate the article in a broader context. | This is the first study, to our knowledge, of opioid drug promotion to nurses in any jurisdiction. This reflects the paucity of literature on nurse-industry interactions in general and the pervasive assumption that non-prescribers are not targets of marketing.^18^ We have posited some hypotheses in the background section that draw from the literature on pharmaceutical industry promotion that suggests a key aim for promotion is to expand the indications for a drug, its use in different populations, and to increase assessment/diagnosis of conditions that the drug can treat, which may implicate nurses (and other health professionals) in promotional efforts. We justify the focus on nurses due to their presence across all healthcare settings, their leadership in community and long term care settings, and emerging evidence that they are an important audience for promotion. | The revised text in the section on “Opioid promotion to nurses” now reads,  “Through promotional campaigns, pharmaceutical companies seek not only to increase their market share, but also to expand their market through promoting awareness, screening, assessment of conditions for which the drug is indicated and expanding the indications for the drug, particularly to new settings and populations ^3^ Nurses play a key role in assessing pain, and administering opioid medications and particularly, medications prescribed ‘as needed,’ across multiple healthcare settings including acute care, primary care, and long term care. Nursing practice also attends to the multiple psychosocial factors that serve as determinants of pain and they play a large role in coordinating care, referrals, and the administration of alternative modalities for pain treatment ^4,5^. Thus, it is important to understand patterns in promotion targeted at nurses as well as prescribers, particularly as nurses’ relationships with industry have received less policy attention than physicians’. |
| Beyond payment reporting, how are drug company interactions with nurses regulated in Australia, i.e. professional associations, hospitals, government regulation? | Like physicians, nurses’ relationships with industry are primarily governed by the industry Code of Conduct overseen by Medicines Australia, the industry trade association. Internationally, nurses’ relationships with industry have received little policy attention and thus, there is little guidance from professional associations, the health system and government in this regard. In many jurisdictions, non-prescribing nurses are excluded from transparency legislation, which makes the Australian data somewhat unique. We have now included some of this detail in the introduction. | The additional material includes,  “Thus, it is important to understand patterns in promotion targeted at nurses as well as prescribers, particularly as nurses’ relationships with industry have received less policy attention than physicians’. In Australia, nurses’ relationships with industry are primarily governed by the industry Code of Conduct ^19^, and there is little guidance specifically for nurses from professional associations, the health system or the government.” |
| Could you provide a bit more context on the opioid product portfolios of the three companies. Are there any significant differences between them? Equally important, to what extent, in your evaluation, do these companies represent opioid drug manufacturers more broadly? It would be helpful to see some commentary on these issues to be able to contextualise the findings. Some of the information on companies could be placed in the introduction; while you mention some of it in the discussion I believe it would be better to start off with a bigger picture of the opioid sector. | We have provided additional details about the included companies’ ownership and product portfolios in the Methods section. We have also made the connection between Purdue Pharma and Mundipharma in the introduction and provided statistics about the uptake of oxycodone in Australia to better illustrate the dominance of this opioid analgesic on the Australian market and to provide a sense of the ‘bigger picture’, which is consistent with data from the United States.  We have also added a paragraph to the Discussion to better contextualise these findings and to caution against over-generalisation. We discuss key limitations of these data including that they are voluntarily reported by members of Medicines Australia, which only represents prescription, brand name manufacturers (and not generic drug manufacturers). Further, membership is voluntary (and Mundipharma has since revoked its membership). | The added sentence in the Introduction now reads,  “Since 2010, prescriptions for OxyContin began to fall in the United States; however, the owners of Purdue Pharma, the Sackler family, pursued similar marketing strategies internationally through a global network of companies, Mundipharma ^1^. In Australia, in 2016-2017, oxycodone (marketed by Mundipharma Australia) accounted for 37% of the 15.4 million opioid prescriptions dispensed ^2^.”  In the Methods, under “Sampling,” we have included details about each of the included companies:  “Thus, we included all events sponsored by:   - Janssen-Cilag Pty Ltd: a subsidiary of Janssen Global, the pharmaceutical branch of Johnson & Johnson, manufactures pharmaceutical products in the areas of mental health, neurology, women health, haematology, gastroenterology, and pain management. - Mundipharma Pty Ltd: founded in 1998, Mundipharma is a privately held company owned by the Sackler family (of Purdue Pharma) and part of the global network of Mundipharma companies, which marketed prescription drugs primarily in the area of pain management, as well as an asthma inhaler and oncology symptom management. - bioCSL / Seqirus Australia Pty Ltd: a member of the CSL Group, a global specialty biotechnology company produces a range of vaccines, antivenoms, and pharmaceuticals related to pain management and allergies; the company is now known as Seqirus”   We have added a paragraph to the Discussion to discuss the representativeness of these data:  “These data provide additional insights into the nature of opioid promotion by prescription, brand name opioid manufacturers, which are consistent with the activities of their multi-national parent companies and subsidiaries. However, sponsorship patterns are not necessarily representative of the activities of opioid manufacturers in Australia, nor internationally, given that data were reported by then-members of Medicines Australia, which is voluntary and does not include the manufacturers of generic drugs, nor others involved in the distribution and dispensing of opioid medications. In the United States, opioid manufacturers and distributors are currently faced with more than 2000 lawsuits alleging that opioid marketing was false and misleading in downplaying the harms and overstating the benefits ^20^. While Purdue Pharma and the other top opioid manufacturers by total sales (including Purdue Pharma, Johnson & Johnson’s Janssen division, Insys, Mylan, and Depomed) have received the greatest public, legal and regulatory scrutiny, federal and state governments have brought legal action against manufacturers of generic opioids (e.g. Endo International, Mallinckrodt Pharmaceuticals and Teva Pharmaceutical Industries), the major distributors (e.g. McKesson, Cardinal Health, and AmerisourceBergen), and a corporate pharmacy chain (Walmart Inc.) ^21^, suggesting that the promotional and commercial origins of the opioid epidemic are highly complex ^20,22,23^.” |
| Please could you provide a bit more information on event descriptions? How extensive were they? Were there any differences in the content or style of descriptions between the included companies? Having been involved in coding of descriptions of payments to patient organisations in the UK I am aware that the specificity and comprehensiveness of payment descriptions can vary considerably, which may have implications for the validity and reliability of coding. It would be good to see some reflection on these points. | As you are clearly well aware, the specificity, comprehensiveness and standardization of the data reporting in these kinds of databases leaves something to be desired!  In some cases, the description of the function contained the name of the drug, the topic of the seminar and its duration (e.g. Janssen Educational Event Series SR Hydromorphone. A once a day oral opioid for chronic pain management - 1 hr). On the other side of the spectrum, the description of the function was sparse and simply stated the event type and duration (e.g. Journal club – 1 hour duration). In all cases, however, the description of the function was accompanied by details on the venue (enabling determination of whether the event was held in a clinical setting or not) and the types and specialties of health professionals attending (which in some cases allowed us to infer the focus, e.g. drug and alcohol specialists, palliative care physicians, or aged care nurses).  We have added some details to the Results section to describe the variability in the reporting. We also note here that there was insufficient information to determine the focus of the event for 32% of the sample.  We note in the limitations section some of the challenges associated with this particular data set. | See additional detail in the Results section in the paragraph first describing Table 2,  “For about a third of events (32%), manufacturers did not report sufficient information to determine the content focus, mentioning only the event type and duration of the event (e.g. “journal club. – 1 hour duration,” “departmental meeting – 2 hour duration”). For the remainder, the description of the event mentioned a topic (e.g. “Palliative care study day), provided the title of a presentation (e.g. “Reducing the burden of persistent pain”), mentioned a particular product (e.g. “Series SR Hydromorphone”), or the focus could be inferred by the venue (e.g. aged care facility) or specialty of professionals in attendance (e.g. orthopaedic surgeons).”  We have added a sentence to the limitations section,  “Further, there was a high degree of variability in terms of the specificity and comprehensiveness of reporting with insufficient detail to determine the content focus of about 1/3 of sampled events.” |
| Could you provide more background on the event types categories? Were these deductive or deductive? If they were taken from manufacturers do they have any standardised descriptions? If the categories were inductive please explain how they were derived. | We adopted the categorization of event types from the study by Fabbri et al (2017), which was a cross-sectional analysis of all sponsored educational events in Australia between 2011 and 2015 (i.e. the entire dataset). Fabbri et al^17^ developed an initial set of keywords for event types based on the literature on industry-health professional interactions and then iteratively, added event type categories to account for all events in the dataset.  We have added some further detail to the Methods to describe this process. | Under Methods, in the “Coding” section, we now add in the first paragraph,  “We coded all events using an iteratively generated keyword search strategy that expanded the coding strategy established by Fabbri et al ^17^. Fabbri et al ^17^ generated keywords based on theoretical variables of interest as outlined in the literature on industry-health professional interactions (e.g. clinical specialty, clinical setting, hospitality provided) and then iteratively expanded the set of keywords within each variable to account for the nature and range of events in the dataset (see Supplementary Table 1).”  And in the final paragraph under “Coding,”  “This coding strategy initially comprised types of events described in the literature on industry-health professional interactions and then iteratively added keywords to account for all event types in the dataset ^17^.” |
| Did you consider conducting a separate set of analyses for each company and perhaps trying to interpret it in the context of its product portfolio? | We elected not to make any formal or statistical comparisons among the three companies as the number of pain-related events sponsored by each company varied so widely, bioCSL sponsored only 15 events, and the sample was dominated by Mundipharma-sponsored events.  We have however, added more detail regarding each sampled company’s ownership and product portfolio to the Methods section under “Sampling.”  Although it might be reasonable to assume that the number of sponsored events reflects the importance of opioid medications within each company’s portfolio, we do not feel that we have sufficiently representative data to support this interpretation, particularly as sponsored events are only one facet of a promotional campaign. We have added a paragraph to the Discussion to further elaborate on this point. | Under Sampling in the Methods, we add:  “Thus, we included all events sponsored by:   - Janssen-Cilag Pty Ltd: a subsidiary of Janssen Global, the pharmaceutical branch of Johnson & Johnson, manufactures pharmaceutical products in the areas of mental health, neurology, women health, haematology, gastroenterology, and pain management. - Mundipharma Pty Ltd: founded in 1998, Mundipharma is a privately held company owned by the Sackler family (of Purdue Pharma) and part of the global network of Mundipharma companies, which marketed prescription drugs primarily in the area of pain management, as well as an asthma inhaler and oncology symptom management. - bioCSL / Seqirus Australia Pty Ltd: a member of the CSL Group, a global specialty biotechnology company produces a range of vaccines, antivenoms, and pharmaceuticals related to pain management and allergies; the company is now known as Seqirus”   The added paragraph in the Discussion reads,  “These data provide additional insights into the nature of promotion of brand name, prescription opioids through events targeted at health professionals, which appear consistent with the activities of manufacturers’ multi-national parent companies and subsidiaries globally ^1,11,20^. However, these data represent what is likely a fraction of total promotional spending: 2012 data from the United States found that sponsorship of educational events accounted for 8% of total marketing expenditure, while detailing (face-to-face visits from sales representatives) accounted for 55% ^24^. That said, this dataset is unique in that expenditures included sponsorship, hospitality, speakers’ fees, and travel reimbursement, so may account for spending beyond what is typically considered “event sponsorship.” |
| **Reviewer 3** | | |
| In this cross-sectional descriptive analysis of pharmaceutical company reports, Dr. Grundy et al examined opioid-related industry educational events for physicians, nurses, and other healthcare professionals from 2011-2015 in Australia.  This is a very important issue, and there are not many data sources that capture industry promotion to non-prescribers (e.g. RNs or pharmacists). Dr. Grundy et al use industry reports, which seem to be limited in details (e.g. not a ton of information presented about the content of these events, and no available breakdown about how many physicians, nurses, or other professionals were at each event).  My main suggestion is that the research question could be more crisply articulated; a lot of characteristics of these events are presented, but the authors could be more explicit about the research question, whether these results are surprising, and what are the direct clinical or policy implications of these data. I have some additional major and minor comments/suggestions below. | Thank you for your constructive comments. We have addressed each point below. We have also tried to clarify and specify the main aim and contribution of the study up front. | The revised aim at the end of the Introductory section reads,  “Thus, we aimed to analyse the nature of pain-related educational events sponsored by opioid manufacturers to understand patterns in relation to the content and setting, and to compare events targeting physicians and nurses. While this analysis can provide historical insight into patterns of opioid prescribing and use in Australia, it also provides a snapshot of the ways that health professional education may serve as a facet of drug promotion more broadly.” |
| - “Coding” para 1 – the authors state “We coded all Mundipharma events as “pain-related” unless the event explicitly stated otherwise, given the company’s limited product profile.” Looking at their website [mundipharma.com.au](http://mundipharma.com.au), it seems they also make eye drops, naloxone, fluticasone/eformoterol inhaler, and a bunch of oncology drugs, as well as buprenorphine (which is not included per Methods) – so I’m not sure this is a valid assumption. The name-brand inhaler and oncology agents could be heavily marketed, as could naloxone… | As we explain in detail above in response to Reviewer 1, Mundipharma had very few non-opioid medications available on the Australian market prior to September 2018 (to account for marketing activities for drugs in the pipeline). These included the inhaler you mention, as well as a few oncology drugs related to lymphoma and symptom management. We developed a set of keywords to account for all events sponsored by Mundipharma that may have been related to other products and applied these to both the description of the event and the professional status of attendees (see Supplementary Table 1). We excluded all events that may have been remotely related to anything other than pain. Otherwise, Mundipharma’s product portfolio during the study period was dominated by opioid medications.  The naloxone medication (Nyxoid) was not given market authorization nor was subsidized until after the study period and is an over-the-counter, pharmacist only medication (and thus is not included in the transparency requirements). Further, we coded for all keywords related to naloxone and opioid overdose reversal and found no events related to these keywords.  We have clarified the text in the Methods to describe this coding process in greater detail.  We note that we may have overestimated the number of pain-related events sponsored by Mundipharma using this strategy. However, given that the promotional activities (both overt and covert) of Mundipharma companies globally are well-documented, we believe this is a reasonable assumption. We have noted this in the Limitations section. | The revised text in the Methods under “Coding” reads,  “We coded all Mundipharma events as “pain-related” unless the event explicitly stated otherwise, given the company’s limited product profile and the dominance of opioid medications within this portfolio. We further assumed that events were “pain-related” in the absence of details about the event content (e.g. 1 hour Journal club) given that Mundipharma is a member of the global network of Mundipharma companies (which are 100% owned by the Sackler family, the owners of Purdue Pharma); Mundipharma’s aggressive promotion of opioid medications and particularly oxycodone internationally is well-documented ^1^. We conducted additional keyword searches to exclude events as “not pain-related” based on Mundipharma’s non-opioid product offerings, which at the time of the study, included respiratory products for asthma, diabetes products, and oncology products related to lymphoma and anti-nausea agents (see Supplementary Table 1).”  We have added text to the Limitations section, which reads,  “We may have overestimated the number of events that were “pain-related” and sponsored by Mundipharma in assuming that events were pain-related in the absence of details about the event content or attendee specialty. However, given that Mundipharma’s product portfolio is dominated by pain-related medications and the promotion of opioid medications by Mundipharma companies both overtly and covertly is well-documented, we believe this was a reasonable assumption ^1^.” |
| - I think the decision not to separately code NPs needs a little more justification, since the paper’s core objective is describing the difference between prescriber-oriented and nursing-oriented educational events. If some of the nurses at “events with at least one nurse” were actually NPs, wouldn’t those events be miscategorized in the analysis (i.e. they were actually prescriber-only)? | The main aim of this study was to compare the nature and extent of promotion of opioids in the form of sponsored educational events to physicians vs nurses. Our interest extends beyond the prescriber/non-prescriber dichotomy to potential disciplinary and practice setting differences and to broaden the discussion about pharmaceutical industry promotion to a multi-disciplinary audience. We have thus clarified the study aim.  Due to the limitations of this data set, we cannot determine the number or proportion of NPs in attendance at an event, only whether they were present or absent. In previous analyses of transparency databases in Australia, we found that companies routinely failed to distinguish between registered nurses and nurse practitioners, referring to all as ‘nurses.’ Thus, it is likely that even if we coded for NPs in attendance, that this would be an underestimate.  To address the concern that events may be miscategorized as including “at least one (non-prescribing)” nurse, we coded the data for all instances where at least one nurse practitioner (NP) was in attendance, which is the category of registered nurses (RNs) in Australia with prescriptive authority. There were 31 events in the entire dataset that included at least one nurse practitioner (31/10,690, 0.3%). There were 20 pain-related events that included at least one nurse practitioner (20/3,411, 0.5%). There was 1 event for nurse practitioners only and 3 that included nurse practitioners and physicians, but no other nurses.  Though NPs in Australia have a similar scope of practice to NPs in the United States (US), NPs in Australia are much fewer in number (1,319 in Australia vs 136,060 in US) and represent a smaller proportion of all RNs (representing about 0.005% of all Australian RNs vs about 5% of US RNs)^25^.Thus, we have elected not to analyse nurse and nurse practitioner-targeted events separately, inferring that registered nurses make up the largest proportion of nurse attendees. | In the Methods, under the section, “Coding,” the revised paragraph now reads,  “We elected not to analyse events for nurses with and without prescriptive authority separately, inferring that non-prescribing nurses are routinely included in pharmaceutical industry-sponsored events in Australia. In Australia, Nurse Practitioners (NPs) are a sub-category of registered nurses (RNs) who have prescriptive authority. In a previous analysis of transparency reports detailing payments from Australian pharmaceutical companies to individual health professionals, we found that companies did not always distinguish between RNs and NPs, referring to individuals as “nurses” ^26^. However, nurses with an NP license likely account for a minority of nurses in receiving industry payments or attending sponsored events. Though NPs in Australia have a similar scope of practice to NPs in the United States (US), NPs in Australia are much fewer in number (1,319 in Australia vs 136,060 in US) and represent a smaller proportion of all RNs (representing about 0.005% of all Australian RNs vs about 5% of US RNs) ^25^. From September 1st 2010, nurse practitioners were authorised to prescribe the opioid medications under study, though their prescribing was limited by scope of practice, state and territory prescribing rights, and contingent on the NP having a collaborative arrangement in place with a physician ^27^.” |
| - It is a limitation that the data does not specify how many of each profession were present at each event (this is stated in the discussion, but may be helpful to include earlier, in the methods). I think the authors did they best they could to offset this, by examining physician-only and nursing-only events separately, but nursing-only events are few, and it’s hard to know what to make of the mixed-profession events, without more context or breakdown of professions in attendance | We have found that the major limitation of this data set is that companies did not specify how many of each profession were present at each event. We note this limitation in the limitations section. The reporting requirements in Australia have since changed and now report payments to individual health professionals rather than groups of health professionals at educational events.  To attempt to provide a clearer picture of the make-up of these events, we have provided some additional detail in the results section. For example, we note that of the multidisciplinary events, those comprising physicians and nurses only account for 55%, thus events can be characterized as truly multidisciplinary and inclusive of multiple health professions. | We have added additional detail to the 3^rd^ paragraph of the Results, which reads,  “Events with attendees from multiple professions were highly multidisciplinary and inclusive: 54% (1066/1964) included only physicians and nurses, while the remainder also included pharmacists (673/1964, 34%), physiotherapists (151/1964, 8%), psychologists (86/1964, 4%), occupational therapists (56/1964, 3%), social workers (39/1964, 2%), dieticians (22/1964, 2%), and various categories of administrative, health system, and research staff.” |
| - There are not very many RN-only events, and I don’t have total clarity about the mixed MD/nursing events (defined as “at least one nurse”; is it just a few nurses or substantial representation? Are we sure that those nurses are non-prescribers, and not NPs?) | Due to the limitations of this data set, we are unable to determine whether the event included just a few nurses or substantial representation. We have noted this in the limitations section.  Similarly, we cannot determine whether the “nurses” in attendance are non-prescribers. However, we have added additional detail about the events where nurse practitioners were explicitly in attendance, finding 4 pain-related events where this was the case (and 1 where nurse practitioners were exclusively in attendance). | See changes to the Methods section under “Coding” and details regarding the professional status of attendees in the 3^rd^ paragraph of the Results,  “The majority of pain-related events were multidisciplinary, with at least one nurse in attendance (1,964/3,411; 58%); 19 multidisciplinary events explicitly included nurse practitioners. Physicians were the only professionals in attendance at 38% of events (1,281/3,411); these events exclusively comprised physicians across specialties and all levels of training. Nurses were exclusively present at 5% of events (166/3,411), one of which included nurse practitioners only.” |
| Results: I also am not sure that these events (>40% journal clubs) are where most of the educational marketing spending is occurring (just $7M over 5 years); does Australia allow door-to-door “educational” detailing in clinical settings, and is it possible that constitutes more of the marketing budget? | As you rightly note, there are numerous forms of pharmaceutical promotion that are currently permitted in Australia including detailing, journal advertisements, sponsorship of patient groups and professional associations, and direct payments to healthcare professionals for research, consulting, and advisory board membership. While there is no publicly available data on marketing spend related to detailing or advertising, at the time of the study, Medicines Australia also required disclosure of sponsorship of patient groups and certain payments to healthcare professionals.  In the last year of this study period (October 2014–September 2015), the total spend by member companies totalled $A89 658 566. The majority of this (83%) was spent on educational events run by the company or third party; 10% in sponsorship of patient groups, and 8% on payments to consultants and advisory board members.^28^  Data from the United States at the time of the study suggest that detailing makes up over half of pharmaceutical marketing expenditures (55%, $15 billion of $27 billion), whereas sponsorship of educational meetings comprises about 8% ($2.1 billion of $27 billion); note, that in the United States, spending on direct-to-consumer advertising, which is not permitted in Australia, is part of this total.^24^  We have added some detail to describe the role of sponsored education within promotional campaigns more broadly. | The revised Discussion paragraph reads,  “These data provide additional insights into the nature of promotion of brand name, prescription opioids through events targeted at health professionals, which appear consistent with the activities of manufacturers’ multi-national parent companies and subsidiaries globally ^1,11,20^. However, these data represent what is likely a fraction of total promotional spending: 2012 data from the United States found that sponsorship of educational events accounted for 8% of total marketing expenditure, while detailing (face-to-face visits from sales representatives) accounted for 55% ^24^. That said, this dataset is unique in that expenditures included sponsorship, hospitality, speakers’ fees, and travel reimbursement, so may account for spending beyond what is typically considered “event sponsorship.” |
| Discussion: I appreciated the point at the end of Para 1 – cancer pain is a stronger indication for opioids, but less featured in educational events. In my mind, the 2 most important conclusions to highlight from these data are that 1) opioid marketing to non-prescribers must be considered, and 2) there is still significant opioid marketing around chronic pain, for which it is not evidence-based | We have bolstered the first paragraph of the Discussion to better highlight the two important conclusions you have suggested. | The end of the first paragraph of the Discussion now reads,  “Thus, industry-sponsored educational events may have focussed on content that was not evidence-based (i.e. the long-term use of opioids for chronic non-cancer pain) and routinely included healthcare teams, including non-prescribing nurses. In examining the role of pharmaceutical industry promotion as a precursor to the inappropriate and harmful prescribing of medications more generally, these data suggest that the “educational” nature of sponsored events should be called into question and that policy efforts to ensure the quality use of medicines should be inclusive of non-prescribers and nurses in particular.” |
| Discussion: I think the authors’ conclusion that educational activities may still be promotional in nature is very astute. It could be more clearly supported by the data (e.g. more explicit examples of the case of oxycodone/naloxone and constipation) | We have added a few examples from the dataset to the paragraph in question to explicitly highlight the promotional intent of the “educational" events described. | The revised text reads,  “In the context of the literature, these results thus suggest that industry-sponsored education may further promotional aims. In the small minority of cases, such as the 248 “product launch” events (e.g. “3 course meal with alcohol/non- alcoholic beverages, Janssen Educational Event: Series SR Hydromorphone. A once a day oral opioid for chronic pain management”) or the 73 events that promoted a brand name drug (e.g. Dinner 3 course, "Presenting TARGIN© Tablets Launch Meeting", Mundipharma has sponsored a General Practitioner Network and had no involvement in inviting attendees or organising the educational component. 2 hour duration”), the promotional intent was explicit. Where detailed, these events (sponsored by Mundipharma) described dinner events where the company’s new brand name product was presented as a solution for chronic pain and opioid-induced constipation (e.g. Dinner 3 course, alcohol provided, CME Meeting: Chronic Pain & Constipation (with NORSPAN© patch). 2 hour duration”), which is consistent with the company’s broader promotional campaigns in Australia during the study period ^29^.” |
| - I wasn’t familiar with bioCSL/CSL or Mundipharma – it may be worth providing a little context about these companies in the intro (I didn’t realize until the discussion that Mundipharma is owned by the Sackler family, e.g.) | We have now made the connection between Mundipharma and Purdue Pharma on the first page in the Introduction. We have also added details about each of the included companies in the Methods section related to ownership and product portfolios. | The added sentence in the Introduction now reads, “  “Since 2010, prescriptions for OxyContin began to fall in the United States; however, the owners of Purdue Pharma, the Sackler family, pursued similar marketing strategies internationally through a global network of companies, Mundipharma ^1^. In Australia, in 2016-2017, oxycodone (marketed by Mundipharma Australia) accounted for 37% of the 15.4 million opioid prescriptions dispensed ^2^.”  In the Methods, under “Sampling,” we have included details about each of the included companies:  “Thus, we included all events sponsored by:   - Janssen-Cilag Pty Ltd: a subsidiary of Janssen Global, the pharmaceutical branch of Johnson & Johnson, manufactures pharmaceutical products in the areas of mental health, neurology, women health, haematology, gastroenterology, and pain management. - Mundipharma Pty Ltd: founded in 1998, Mundipharma is a privately held company owned by the Sackler family (of Purdue Pharma) and part of the global network of Mundipharma companies, which marketed prescription drugs primarily in the area of pain management, as well as an asthma inhaler and oncology symptom management. - bioCSL / Seqirus Australia Pty Ltd: a member of the CSL Group, a global specialty biotechnology company produces a range of vaccines, antivenoms, and pharmaceuticals related to pain management and allergies; the company is now known as Seqirus” |
| - Abstract results – “the majority…were focused on chronic pain (26%)” – I would instead say that chronic pain was the most common topic, followed by XYZ | We have amended the sentence in the Abstract results as suggested. | The revised sentences in the results section of the Abstract reads,  “The majority of events were held in clinical settings (61%) and 43% took the form of a journal club. Chronic pain was the most common event topic (26%) followed by cancer pain and palliative care (18%), and then generic or unspecified references to pain (15%); nearly a third (32%) of event descriptions contained insufficient information to determine the content focus.” |
| - Would be interesting to hear what other multidisciplinary groups were represented at these educational events – e.g. pharmacists? Hospital administrators? PAs and NPs? | We have further coded the multidisciplinary events to provide greater detail regarding the disciplinary composition of attendees. | We provide further detail related to the nature of multidisciplinary events in the 3^rd^ paragraph of the Results section,  “Events with attendees from multiple professions were highly multidisciplinary and inclusive: 54% (1066/1964) included only physicians and nurses, while the remainder also included pharmacists (673/1964, 34%), physiotherapists (151/1964, 8%), psychologists (86/1964, 4%), occupational therapists (56/1964, 3%), social workers (39/1964, 2%), dieticians (22/1964, 2%), and various categories of administrative, health system, and research staff.” |
| - Intro line 5 – what is the unit when you say “the rate of death increased from 1 in 1999 to 4.4 in 2016”? | The unit is per 100,000 standard population.  However, in re-writing the introduction, the sentence in question has now been deleted. | The sentence has been deleted. |
| - The intro doesn’t mention Australia until page 3 – may frame the discussion for Australian data earlier | We have significantly revised and restructured the Introduction in order to frame the focus on Australia up front. We highlight that Australia, like Canada and the United States, faces a similar public health crisis in relation to the harms of opioid use. We also further justify the focus of the study by describing the (globally) unique data source available in Australia that allows for study of the nature of educational events sponsored by opioid manufacturers. | Please see the revised introduction. |
| - Intro page 2 line 4 – would also include industry-sponsored detailing visits and meals as a very common “educational” activity | While we certainly agree with this point, what we attempted to convey in this sentence (which reflects the accompanying citation) was that marketing also occurs through activities that are not typically recognized as “promotional.” We have revised the sentence to clarify this.  We have also added an additional sentence and citation to discuss the ubiquity of meals at “educational” events. | The revised sentence now reads,  “Analysis of internal industry documents suggests that a key pharmaceutical industry marketing strategy is coordinated “education” campaigns comprised of a number of activities not typically recognized as ‘promotional’ including use of key opinions leaders, peer selling, and industry sponsorship of continuing education ^30,31^.”  The additional sentence reads,  “Despite being characterized as “educational,” industry-sponsored events are almost always associated with other payments and/or gifts: food and drink were provided at over 90% of pharmaceutical-industry sponsored educational events for health professionals in Australia between 2011 and 2015 ^17^.” |
| - Typo intro p2 para 2 – “analyses…suggests” | We have corrected the typo noted. | The revised sentence in the introduction (page 2, paragraph 2) reads,  “Analyses of United States data suggest. . . “ |
| - Introduction, End of page 5 – might clarify or flip this sentence (payments associated w/ increased mortality, rather than the other way around) | We have clarified the sentence in question and reordered the clauses as suggested. | The sentence (end of page 5) now reads,  “Analysing the impact of these payments at the county level per 1000 county population, researchers found that increases in the number of payments to physicians, the number of physicians receiving marketing, and the total monetary value of payments to physicians were associated with increased morality from opioid overdoses.” |
| - Introduction, This sentence could be clarified: “In Australia, nurses attended 40% of pharmaceutical-industry sponsored educational events at twice the rate of primary care physicians [15].” | We have reworded the sentence for clarity. | The sentence now reads,  “In Australia, 40% of pharmaceutical-industry sponsored educational events included nurses; in contrast, 21% of sponsored events included primary care physicians ^32^.” |
| - Methods, Would include a sentence of explanation why you excluded meds for breakthrough cancer pain | We have added a sentence to the Methods under “Sampling” to explain our decision to exclude opioid medications for breakthrough cancer pain.  Our sample is purposive in that we aimed to examine the nature and implications of promotion of opioid medications in the form of ‘educational events.’ Thus, we included companies and medications that were likely to be promoted in this format during the study period and potentially for indications or populations that carry the risk of public health harm.  We found quite a few companies that manufactured opioids for breakthrough cancer pain, however all had very low usage compared to other opioids.^10^ For example Astrazeneca manufactured PecFent (fentanyl nasal spray), Takeda manufactured Instanyl (also fentanyl nasal spray), Menarini Australia made Abstral (fentanyl lozenges) and also nasal spray, Teva made fentanyl lozenges. PecFent and Instanyl have since been removed for the market (see Table 1 in the manuscript).  In all cases, the medication for breakthrough cancer pain was the only opioid the company manufactured and exploratory searches of the dataset found that pain-related educational events were a tiny fraction of the events sponsored by the company and likely related to other medications in their portfolio. | The sentence added to the Methods reads,  “Manufacturers of opioids whose primary indication is anaesthesia, substance use disorder treatment, or breakthrough cancer pain as these products have very low usage in comparison with other opioids ^10^; in all cases, companies that manufactured opioids for these indications marketed only one product and had few to no pain-related events.” |
| - Results, Para 3 (describing the cost range between events) was a little hard to follow | We have re-worked this paragraph (4^th^ paragraph of the Results) for clarity. | The revised paragraph now reads,  “Table 2 details the characteristics of pain-related events. The three opioid manufacturers spent more than AUD 7.6 million on pain-related events; however, the median cost per attendee was nominal (approximately AUD 15). Food and beverage costs accounted for about a third of the total costs, at just under AUD 2.5 million overall. Physician-only events accounted for about half of the total spend (AUD 3,962,226, 52%). The most expensive event (at AUD 350,000) was for physicians only, however physician-only events also had the largest range in event costs. Spending on nurse-only events was proportionate to the number of nurse-only events. In contrast to physician-only events, the most expensive nurse-only event was AUD 16,824, however, nurse-only events had the highest median cost per event at approximately AUD 930, which equalled to a median of approximately AUD 27 per attendee.” |
| - Results, Table 2 – would include median cost per attendee. Also curious what the non-clinical settings were? | We have added a row to Table 2 detailing the median cost per the median number of attendees per event. Please note that there were no significant differences among attendee groups.  We have added a footnote to Table 2 providing some examples of non-clinical settings. These were largely restaurants and hotels, but also included convention centres, community centres and wineries. | See Table 2, row “Median cost/ median number of attendees per event.”  See Table 2, footnote “f”, which reads,  “Non-clinical settings largely included restaurants, hotels, and convention centres.” |
| - Discussion, Para 2 – what do you mean by oxycodone/naloxone’s “subsidy”? I wonder if increased uptake among older adults was due to increased constipation in this population | Australia has a federal government program, the Pharmaceutical Benefits Scheme, which subsidizes prescription medications for all citizens and residents. For adults over the age of 65, the subsidy is higher and thus, older adults pay a lower co-payment than general beneficiaries.  In December 2011, coinciding with the beginning of this study’s period, oxycodone/naloxone CR marketed by Mundipharma was approved for subsidy under the Pharmaceutical Benefits Scheme for treatment of chronic severe disabling pain, the same indication as the single-ingredient oxycodone CR.^29^ In 2014, (the end of this study period), the single ingredient oxycodone CR (>10mg) was reformulated to be tamper resistant and the 5mg formulation withdrawn. Thus, as of 2014 the oxycodone/naloxone CR formulation was the only 5mg formulation on the Australian market.^29^  Though it is unknown to what extent the subsidy, marketing efforts, or actual incidence of constipation among older adults contributed to the increased uptake of this drug, cross-national comparisons provide some insight. Although the United States FDA approved the oxycodone/naloxone CR formulation in 2014 and gave it an abuse-deterrent label, the drug was never marketed.^29^ In Canada, the drug was approved, but there is no public drug subsidy, so uptake has been limited. Further, in North American promotion has focused on the drug’s abuse-deterrent properties.^29^ The promotion of the drug as a solution to opioid-induced constipation appears to be somewhat unique to Australia, while the incidence of constipation among older adults does not.  We have amended the sentence in question to clarify the meaning of “subsidy” and to provide further relevant detail. | We have revised the second paragraph of the Discussion to read,  “Coinciding with the study period, there was rapid uptake of Mundipharma’s oxycodone/naloxone controlled-release product in Australia with promotion focused on its beneficial effects on opioid-induced constipation ^29^. Australia has a federal government program, the Pharmaceutical Benefits Scheme, which subsidizes prescription medications for beneficiaries. An analysis of dispensing claims from 2006-2016 found that, following approval of the 5 mg oxycodone/naloxone controlled-release formulation for subsidy under the Pharmaceutical Benefits Scheme, dispensing claims grew 1.6-fold for oxycodone controlled-release dispensing, driven entirely by rapid uptake of the low strength (≤5 mg) oxycodone/naloxone controlled-release formulation ^29^. Uptake of the drug was greater than expected if it were substituting the single-ingredient oxycodone controlled release, suggesting instead, an expansion of the oxycodone controlled-release market ^29^. Uptake was greatest among older individuals initiating opioid treatment, despite recommendations against initiation with a long-acting formulation due to increased risk of adverse effects such as accidental overdose and respiratory depression ^29^. |
| - Discussion, Para 4 suggests that opioid events targeted more multidisciplinary groups, but the same # of nurses. Was it other professions driving the multidisciplinary nature of opioid events (eg pharmacists?) | Within the dataset, 15% (1573/10690) events included at least one pharmacist. Researchers have previously analysed the nature of industry-sponsored events targeted at pharmacists in Australia.^33^ Thus, we elected to focus on nurses as this is a gap in the literature. Further, nurses attended nearly half of the events in the dataset (47%, 5,044/10,690).  We further coded the multidisciplinary events to provide greater detail regarding the disciplinary composition of attendees. Just over half of multidisciplinary events (54%) included physicians and nurses only. Due to limitations with the dataset, there is no way to determine how ‘multidisciplinary’ these events were as we could not distinguish events with just one nurse from those with substantial nursing representation.  The other half of multidisciplinary events included a wide range of professions and members of the healthcare team including pharmacists (34% of multidisciplinary events), and also physiotherapists, psychologists, occupational therapists, social workers, and various categories of staff (all <10% of multidisciplinary events). Thus, these events can be characterized as inclusive of healthcare teams. We have added these insights to the paragraph in the Discussion to suggest that non-industry sponsored education and also efforts to regulate promotion in clinical settings should adopt a similarly inclusive and multidisciplinary approach. | We have added text to the 4th paragraph of the Discussion, which reads,  “Although most multidisciplinary pain-related events (54%) included physicians and nurses only, many were highly multidisciplinary, including a wide range of health professionals and members of the healthcare team, suggesting that promotional efforts are inclusive. Initiatives such as education or policy designed to promote the safe and quality use of opioid medications should adopt a similarly multidisciplinary and inclusive approach.” |
| - Discussion, Would be cautious of the statement that “nurses attended product lunches more frequently than groups including physicians,” since there were 248 such events for physicians-only and 28 product launches for nurses-only | We have deleted this sentence for accuracy and edited the previous sentence to indicate that nurses, too, are the target audience for promotional events such as product launches. | We have revised the first sentence of the 5^th^ paragraph of the Discussion to read,  “Events, including product launches, were also targeted specifically at nurses without prescriptive authority, indicating that non-prescribers are a target audience for pharmaceutical industry sponsorship beyond being collateral attendees at physician-focused events.” |
| - Discussion, Para 5 and 6 may overstep the data somewhat – not sure it can be extrapolated from the differences in Table 2 that “this type of promotion may reflect the influence of nursing in community, home and long-term care settings and create risk for misuse of opioids in vulnerable populations”. Could it be argued that nursing education improves safety or control of hospice & cancer pain, or that some of these Mundipharma trainings were in reference to rescue use of Narcan (naloxone)? | As we mention above, we are confident that these trainings were not related to the use of naloxone as Mundipharma did not have a naloxone preparation on the market during the study period, nor did we find any events that matched keywords related to naloxone or overdose reversal.  However, we agree that this interpretation extrapolates beyond the available data. Thus, we have deleted the sentence in question and suggested that this may be an important avenue for future work given Mundipharma’s more recent efforts targeting nurses. | The revised text now reads,  “The extent and impact of opioid promotion to nurses across healthcare settings remains underexplored and is an important avenue for future research. For example, following the study period, Mundipharma launched educational campaigns designed to facilitate their implementation in long term care settings (see “PAN.AC.EA,” or, “Pain Advocacy Nurse in Aged Care for Education & Assessment ^34^). |
| - Discussion, Is it really true that non-industry education about opioids has not been effective? Is there a review (eg Cochrane) that you could cite? | We cited two Australian trials, which found no significant effect of an educational intervention on prescribing habits or intent. However, we have revised this paragraph to include a recent systematic review and meta-analysis of North American studies that evaluated interventions to improve prescribing for chronic non-cancer pain, including education ^35^. They found very low-certainty evidence that education improved the uptake of other interventions recommended in clinical practice guidelines. | The revised text in the last paragraph of the Discussion reads,  “However, this also raises the question of who should be responsible for delivering education about pharmaceuticals to healthcare professionals and suggests pragmatic challenges as industry-independent educational interventions are in no way comparable in terms of resources, reach and duration to industry-sponsored educational events. Two Australian trials examined the effects of independent chronic pain management education programs among general practitioners and found no significant effect on prescribing habits or intent ^36,37^. However, a systematic review and meta-analysis of interventions to improve prescribing practices for chronic non-cancer pain found very low-certainty evidence that education could indirectly improve prescribing practices through increased uptake of treatment agreements, urine drug testing, and use of prescription drug monitoring programs ^35^. Thus, the question is perhaps not how to deliver ‘counter’ education, which may not be effective on its own in mitigating the harms associated with industry-sponsored education, but how it might be incorporated into multi-faceted ^35^, multidisciplinary, and structural efforts to improve the quality use of medicines and reduce opioid-related harms.” |
| - Discussion, I appreciate the point that even pharmaceutical “educational” events should be regulated as promotion. There are questions that arise as to who would deliver education if Pharma did not (e.g. without these industry activities, would it take longer for an effective new drug to reach prescribers & patients – even if that info is favorably biased) | We agree that this is an important question and one frequently raised following critiques of industry-sponsored education. We have now raised this question in the text. However, in light of a recent systematic review and meta-analysis of interventions to improve prescribing for chronic non-cancer pain, we suggest that the question is not who should deliver education, but what the role of education is in terms of improving prescribing practices. We suggest instead that education be an adjunct to more structural reforms. | The revised text in the last paragraph of the Discussion reads,  “However, this also raises the question of who should be responsible for delivering education about pharmaceuticals to healthcare professionals and suggests pragmatic challenges as industry-independent educational interventions are in no way comparable in terms of resources, reach and duration to industry-sponsored educational events. Two Australian trials examined the effects of independent chronic pain management education programs among general practitioners and found no significant effect on prescribing habits or intent ^36,37^. However, a systematic review and meta-analysis of interventions to improve prescribing practices for chronic non-cancer pain found very low-certainty evidence that education could indirectly improve prescribing practices through increased uptake of treatment agreements, urine drug testing, and use of prescription drug monitoring programs ^35^. Thus, the question is perhaps not how to deliver ‘counter’ education, which may not be effective on its own in mitigating the harms associated with industry-sponsored education, but how it might be incorporated into multi-faceted ^35^, multidisciplinary, and structural efforts to improve the quality use of medicines and reduce opioid-related harms.” |

**References**

1.	Ryan H, Girion L, Glover S. OxyContin goes global — “We’re only just getting started”. *LA Times.* December 18, 2016, 2016.

2.	Australian Institute of Health and Welfare (AIHW). *Opioid harm in Australia and comparisons between Australia and Canada.* Canberra, Australia2018.

3.	Moynihan R, Cassels A. *Selling sickness: How the worlds' biggest pharmaceutical companies are turning us all into patients.* New York, NY: Nation Books; 2005.

4.	American Nurses Association. *The opioid epidemic: The evolving role of nursing.* Silver Spring, MD: American Nurses Association;2018.

5.	ANA Center for Ethics and Human Rights. *The ethical responsibility to manage pain and the suffering it causes.* Silver Spring, MD: American Nurses Association;2018.

6.	Berterame S, Erthal J, Thomas J, et al. Use of and barriers to access to opioid analgesics: a worldwide, regional, and national study. *The Lancet.* 2016;387(10028):1644-1656.

7.	Gomes T, Tadrous M, Mamdani MM, Paterson JM, Juurlink DN. The Burden of Opioid-Related Mortality in the United States. *JAMA Netw Open.* 2018;1(2):e180217.

8.	Gelineau K. Opioid crisis goes global as death surge in Australia. *AP News.* September 5, 2019, 2019.

9.	Blanch B, Pearson SA, Haber PS. An overview of the patterns of prescription opioid use, costs and related harms in Australia. *Br J Clin Pharmacol.* 2014;78(5):1159-1166.

10.	Karanges EA, Blanch B, Buckley NA, Pearson SA. Twenty-five years of prescription opioid use in Australia: a whole-of-population analysis using pharmaceutical claims. *Br J Clin Pharmacol.* 2016;82(1):255-267.

11.	Van Zee A. The promotion and marketing of OxyContin: Commercial triumph, public health tragedy. *American Journal of Public Health.* 2009;99(2):221-227.

12.	Dhalla IA, Persaud N, Juurlink DN. Facing up to the prescription opioid crisis. *BMJ.* 2011;343:d5142.

13.	Hadland SE, Krieger MS, Marshall BDL. Industry payments to physicians for opioid products, 2013-2015. *Am J Public Health.* 2017;107(9):1493-1495.

14.	Hadland SE, Cerdá M, Li Y, Krieger MS, Marshall BL. Association of pharmaceutical industry marketing of opioid products to physicians with subsequent opioid prescribing. *JAMA Internal Medicine.* 2018.

15.	Hadland SE, Rivera-Aguirre A, Marshall BDL, Cerda M. Association of pharmaceutical industry marketing of opioid products with mortality from opioid-related overdoses. *JAMA Netw Open.* 2019;2(1):e186007.

16.	Robertson J, Moynihan R, Walkom E, Bero L, Henry D. Mandatory disclosure of pharmaceutical industry-funded events for health professionals. *Plos Med.* 2009;6(11):e1000128.

17.	Fabbri A, Grundy Q, Mintzes B, et al. A cross-sectional analysis of pharmaceutical industry-funded events for health professionals in Australia. *BMJ Open.* 2017;7(6).

18.	Grundy Q. *Infiltrating healthcare: How marketing works underground to influence nurses.* Johns Hopkins University Press; 2018.

19.	Medicines Australia. *Code of Conduct.* Deakin, ACT2019.

20.	Hoffman J. Johnson & Johnson ordered to pay $572 million in landmark opioid trial. *New York Times.* August 30, 2019.

21.	Department of Justice files nationwide lawsuit against Walmart Inc. for Controlled Substances Act violations [press release]. Washington, DC: U.S. Department of Justice2020.

22.	Mangan D. Opioid epidemic: Senate committee opens probe of five big painkiller makers. *CNBC.* March 28, 2017, 2017.

23.	Hoffman J. $260 Million Opioid Settlement Reached at Last Minute With Big Drug Companies. *The New York Times.* October 21, 2019, 2019.

24.	Pew Prescription Project. *Persuading the prescribers: Pharmaceutical industry marketing and its influence on physicians and patients.* Philadelphia, PA: The Pew Charitable Trusts;2013.

25.	Bureau of Labor Statistics. Occupational employment statistics. United States Department of Labor. https://www.bls.gov/oes/current/oes_stru.htm#29-0000. Published 2016. Accessed June 4, 2016.

26.	Karanges EA, Grundy Q, Bero L. Understanding the Nature and Extent of Pharmaceutical Industry Payments to Nonphysician Clinicians. *JAMA internal medicine.* 2019;179(10):1430-1432.

27.	The Pharmaceutical Benefits Scheme (PBS). Nurse practitioner PBS prescribing. The Commonwealth of Australia. https://www.pbs.gov.au/browse/nurse. Published 2020. Accessed January 4, 2021.

28.	Parker L, Karanges EA, Bero L. Changes in the type and amount of spending disclosed by Australian pharmaceutical companies: an observational study. *BMJ Open.* 2019;9(2):e024928.

29.	Schaffer AL, Karanges EA, Buckley NA, et al. Increases in controlled-release oxycodone utilisation following the subsidy of oxycodone with naloxone formulations: An Australian population-based study. *Pharmacoepidemiology and Drug Safety.* 2019;28(1):97-105.

30.	Steinman MA, Bero LA, Chren M-M, Landefeld CS. Narrative review: the promotion of gabapentin: an analysis of internal industry documents. *Ann Intern Med.* 2006;145(4):284-293.

31.	Sismondo S. Key opinion leaders and the corruption of medical knowledge: what the Sunshine Act will and won't cast light on. *J Law Med Ethics.* 2013;41(3):635-643.

32.	Grundy Q, Fabbri A, Mintzes B, Swandari S, Bero L. The inclusion of nurses in pharmaceutical industry–sponsored events: Guess who is also coming to dinner? *JAMA Internal Medicine.* 2016;176(11):1718-1720.

33.	Robertson J, Walkom E, Moynihan R, Bero L, Henry D. Pharmaceutical industry funding of educational events for pharmacists in Australia: an analysis of data from the first 6 months of a mandatory disclosure programme. *International Journal of Pharmacy Practice.* 2010;18(2):88-92.

34.	Mundipharma Pty Ltd. *PAN.AC.EA: Pain Advocacy Nurse in Aged Care for Education & Assessment* Sydney, Australia: Mundipharma Pty Ltd;Undated.

35.	Asamoah-Boaheng M, Badejo OA, Bell LV, et al. Interventions to Influence Opioid Prescribing Practices for Chronic Noncancer Pain: A Systematic Review and Meta-Analysis. *Am J Prev Med.* 2021;60(1):e15-e26.

36.	Holliday SM, Hayes C, Dunlop AJ, et al. Does brief chronic pain management education change opioid prescribing rates? A pragmatic trial in Australian early-career general practitioners. *PAIN.* 2017;158(2).

37.	McCracken LM, Boichat C, Eccleston C. Training for general practitioners in opioid prescribing for chronic pain based on practice guidelines: A randomized pilot and feasibility trial. *The Journal of Pain.* 2012;13(1):32-40.
